# The Brazilian Portuguese version of the revised Maastricht Upper Extremity Questionnaire (MUEQ-Br revised): translation, cross-cultural adaptation, reliability, and structural validation

**DOI:** 10.1186/s12891-015-0497-2

**Published:** 2015-02-25

**Authors:** Aline Mendonça Turci, Débora Bevilaqua-Grossi, Carina Ferreira Pinheiro, Marcela Mendes Bragatto, Thais Cristina Chaves

**Affiliations:** Ribeirão Preto Medical School, University of São Paulo (USP), Ribeirão Preto, SP Brazil; Department of Biomechanics, Medicine and Rehabilitation of the Locomotor System, USP, Ribeirão Preto, SP Brazil; Post Graduate Program in Rehabilitation and Functional Performance, USP, Ribeirão Preto, SP Brazil; Department of Neurosciences and Behavioral Sciences, USP, Ribeirão Preto, SP Brazil

**Keywords:** Computer work, Questionnaire, Validation, Cross-cultural adaptation, Occupational health, Upper extremity

## Abstract

**Background:**

Complaints of the arm, neck, and shoulders (CANS) have a multifactorial etiology, and, therefore, their assessment should consider both work-related ergonomic and psychosocial aspects. The Maastricht Upper Extremity Questionnaire (MUEQ) is one of a few specific tools available to evaluate the nature and occurrence of CANS in computer-office workers and the impact of psychosocial and ergonomic aspects on work conditions. The purpose of the present study was to perform a translation and cross-cultural adaptation of the MUEQ to Brazilian Portuguese and verify the reliability, internal consistency, and structural validity of the MUEQ in Brazilian computer-office workers.

**Methods:**

The cross-cultural adaptation consisted of five stages (forward translation of the MUEQ to Brazilian Portuguese, synthesis of the translation, back-translation, expert committee meeting, and the pre-final-version test). In the pre-final-version test, 55 computer-office workers participated. For reproducibility, a sample of 50 workers completed the questionnaire twice within a one-week interval. A sample of 386 workers from the University of São Paulo (mean age = 37.44 years; 95% confidence interval: 36.50–38.38; 216 women and 170 men) participated on the structural validation and internal consistency analysis. Intraclass correlation coefficient was used for the statistical analysis of reproducibility, Cronbach’s alpha was used for internal consistency, and confirmatory factor analysis was used for structural validity.

**Results:**

The calculation of internal consistency, reproducibility, and cross validation provided evidence of reliability and lack of redundancy. The psychometric properties of the modified MUEQ-Br revised were assessed using confirmatory factor analysis, which revealed 6 factors and 41 questions. For this model, the comparative fit index (CFI), goodness-of-fit index (GFI), and non-normed fit index (NNFI) each achieved 0.90, and the consistent Akaike information criterion (CAIC), chi-square, expected cross-validation index (ECIV), and root mean square error of approximation (RMSEA) demonstrated better values.

**Conclusions:**

The results provide a basis for using the 41-item MUEQ-Br revised for the assessment of computer-office workers’ perceptions of the psychosocial and ergonomic aspects of CANS and musculoskeletal-complaint characterization.

**Electronic supplementary material:**

The online version of this article (doi:10.1186/s12891-015-0497-2) contains supplementary material, which is available to authorized users.

## Background

Complaints of the arms, neck, and shoulders (CANS) are defined as musculoskeletal complaints of the arms, shoulders, and/or neck that are not caused by acute trauma or systemic diseases [1]. In the early 1970s, CANS were acknowledged as the major cause of work-related disabilities [2]. CANS may cause severe and debilitating symptoms, such as pain, numbness, and tingling [2]. The reported prevalence of musculoskeletal complaints among computer-office workers is 10–62% [3], and the most frequent complaints are related to the neck and shoulders [2,4,5].

Over the last 20 years, there has been a significant increase in the number of individuals who use computers at their jobs [6-8]. In developed countries, the percentage of computer-office workers increased from 33% in 1989 to 57% in 2000, with nearly 80% of the workforce using computers on a daily basis [9].

According to reports from developed nations [3], the increase in computer use seems to be related to the development of CANS and cause-effect relationships have been reported in the literature [10]. CANS are also seen as a trait in developing countries [11]. The Brazilian Institute of Geography and Statistics (IBGE) (2012) showed that only 19.5% of companies do not use a computer in their activities, and that 46% of Brazilians have a computer at home [12]. The rapid economic development of recent decades has led to an increase in use of computer systems in state- and private-sector organizations as a way to improve productivity. However, unlike for other developing countries, there are no published data on the extent of work-related CANS in Brazil.

CANS among computer-office workers appear to have a multifactorial etiology [2,3], and a recent overview of systematic reviews [13] reported that the literature supports an association between computer use and musculoskeletal disorders, but does not identify a cause-effect relationship. Thus, multiple factors (e.g., use of a computer per se, time spent using a mouse and keyboard [14], work-station design, and psychosocial factors such as poor support, job strain, and high demand) could all be associated with the clinical features of musculoskeletal disorders and CANS [14]. Wahlstrom [3] proposed a model that sketches the factors contributing to an association between musculoskeletal disorders and computer work, and highlighted the factors of work organization, psychosocial factors, and mental stress.

Thus, a validated instrument that is able to assess both the prevalence of CANS and evaluate its associated factors would be valuable in countries like Brazil where data on CANS is minimal. There are some instruments available in Brazilian Portuguese to assess aspects of work, such as the Quick Exposure Check [15], Job Factors Questionnaire [16], and Nordic Musculoskeletal Questionnaire [17]. However, the Maastricht Upper Extremity Questionnaire (MUEQ) is the only tool available that assesses both physical and biopsychosocial aspects related to CANS in computer-office workers [8,18].

The MUEQ has been cross-culturally adapted to several languages, including Arabic [11], Greek [18], and Sinhalese [19]. However, before these versions are used internationally, they must undergo cross-cultural adaptation and validation processes as suggested by the Consensus-based Standards for the Selection of Health Measurement Instruments (COSMIN) [20].

The objective of the present study was to describe the process of cross-cultural adaptation of the MUEQ to Brazilian Portuguese, and to verify the psychometric properties of the questionnaire (i.e., reproducibility, structural validity, and internal consistency) when applied to Brazilian computer-office workers.

## Methods

### Study population and data collection

This study was conducted from December 2012 to April 2013. Questionnaires were sent to 627 computer-office workers at the University of São Paulo (USP), Ribeirão Preto campus. USP is a public institution, and 51% of the office workers are female. In addition, 24% have a higher-education degree, and 76% have a technical formation or high school degree.

For this project, the computer-office-worker sample consisted of men and women employees who were between the ages of 18 and 60 years, had been in the same job position for at least 12 months, and used a computer for a minimum of four hours each work day [6].

### Sample size for study I: cross-cultural adaptation

This phase included 55 individuals (mean age: 33.56 years, SD: 7.93, 95% CI: 31.48–35.64, 41 women, 14 men) who had worked with a computer at their jobs for an average of 10.40 years (95% CI: 8.31–12.49), been at the same job for 7.08 years (95% CI: 5.23–8.93), and worked an average of 7.21 hours/day using a computer (95% CI: 6.87–7.56). The pre-final version included a pre-test with 15 volunteers (mean age: 31.54 years, SD: 5.08, 95% CI: 28.97–34.11 years, 12 women, and 3 men) who had worked with a computer for an average of 10.27 years (CI 95%: 6.50–14.04).

### Sample size for study II: reproducibility test

The reproducibility test was conducted with 50 workers, with an average age of 36.04 years (SD: 8.46, 95% CI: 33.70–38.38) who had worked using a computer at their jobs for a mean of 12.86 years (95% CI: 10.71–15.01); 22 were women and 28 were men.

### Sample size for study III: validation study

Of the 627 questionnaires handed out, only 386 (participant mean age: 37.44 years, SD: 9.38, 95% CI: 36.50–38.38) were included in the confirmatory factor analysis (CFA) and internal consistency phases. Thus, we obtained a response rate of 62%, and the mean time taken to complete the questionnaire was 14.67 minutes (95% CI: 13.88–15.46). Of the participants, 216 were women and 170 were men (see Table 1), and they had used a computer at their jobs for 13.52 years (95% CI: 12.38–14.36).

**Table 1:** **Characteristics of the sample for the internal consistency and confirmatory factor analyses**

	**Total sample n = 386**	**Prevalence** * **total sample (95% CI)**	**Total female participants n = 216**	**Prevalence of women (95% CI) n = 216**	**Total male participants n = 170**	**Prevalence of men (95% CI)n = 170**
*Age*						
20–30	118	0.31 (0.26–0.35)	56	0.26 (0.21–0.32)	62	0.36 (0.30–0.44)
31–40	115	0.30 (0.25–0.35)	57	0.26 (0.21–0.33)	58	0.34 (0.27–0.42)
41–50	116	0.30 (0.26–0.35)	78	0.36 (0.30–0.43)	38	0.22 (0.17–0.29)
51–60	37	0.10 (0.07–0.13)	25	0.12 (0.08–0.17)	12	0.07 (0.04–0.12)
*Years in current work position*						
1–5	170	0.44 (0.39–0.49)	36	0.17 (0.12–0.22)	40	0.24 (0.18–0.30)
6–10	74	0.19 (0.16–0.23)	44	0.20 (0.16–0.26)	50	0.29 (0.23–0.37)
11–15	48	0.12 (0.10–0.16)	42	0.19 (0.15–0.25)	35	0.21 (0.15–0.27)
>15	94	0.24 (0.20–0.29)	94	0.44 (0.37–0.50)	45	0.26 (0.20–0.34)
*Years working with a computer*						
1–5	76	0.20 (0.16–0.24)	36	0.17 (0.12–0.22)	40	0.24 (0.18–0.30)
6–10	94	0.24 (0.20–0.29)	44	0.20 (0.16–0.26)	50	0.29 (0.23–0.37)
11–15	77	0.20 (0.16–0.24)	42	0.19 (0.15–0.25)	35	0.21 (0.15–0.27)
>15	139	0.36 (0.31–0.41)	94	0.44 (0.37–0.50)	45	0.26 (0.20–0.34)
*Number of hours worked per day*						
6–8	361	0.94 (0.91–0.96)	200	0.93 (0.88–0.95)	161	0.95 (0.90–0.97)
>8	25	0.06 (0.04–0.09)	16	0.07 (0.05–0.12)	9	0.05 (0.03–0.10)
*Number hours working with a computer per day*						
4–6	107	0.28 (0.23–0.32)	46	0.21 (0.16–0.27)	61	0.36 (0.29–0.43)
7–8	263	0.68 (0.63–0.73)	160	0.74 (0.68–0.79)	103	0.61 (0.53–0.68)
>8	16	0.04 (0.03–0.07)	10	0.05 (0.03–0.08)	6	0.04 (0.02–0.07)

*Note.* “CI” = confidence interval and “n” = sample size.

*Prevalence calculation: number of subjects in each interval/total sample size.

The exclusion criteria were: illiteracy or functional illiteracy, visual impairment (not corrected with glasses/contact lenses), and hearing impairment (not corrected with a device). This project was reviewed and approved by the Human Research Ethics Committee of the University Hospital at Ribeirão Preto Medical School, University of São Paulo (Process HCRP N° 10299/2012). All participants signed an informed consent form.

### The MUEQ instrument

The MUEQ addresses the occurrence, nature, and possible work-related physical and psychological factors associated with CANS among computer users. The MUEQ is the result of the combination of previous tools; questions related to psychosocial factors are based on the Job Content Questionnaire, and the questions related to physical factors at work are based on the Dutch Musculoskeletal Questionnaire [21,22].

The original MUEQ consisted of 59 questions, and individuals were allowed 15 minutes to complete it (see Additional file 1). The first part of the MUEQ assesses socio-demographic characteristics. The MUEQ consists of seven basic domains: (l) work station (seven questions), (2) body posture during work (11 questions), (3) job control (nine questions), (4) job demands (seven questions), (5) break time (eight questions), (6) work environment (nine questions), and (7) social support (seven questions).

The work-station domain has seven questions, with a maximal score of 7 points and two response options (“No” = 1 and “Yes” = 0). For all the other domains, the response options were “Always” (3 points), “Often” (2 points), “Sometimes” (1 point), “Seldom” (0 points), and “Never” (0 points). The body-posture-during-work domain ranges from 0 to 33 points, the job-control domain ranges from 0 to 27 points, the job-demands domain ranges from 0 to 21 points, the break-time domain ranges from 0 to 24 points, the work-environment domain ranges from 0 to 27 points, and the social-support domain ranges from 0 to 24 points.

Complaint items that assess the frequency and clinical features of neck and upper-arm complaints are included in the final portion of the MUEQ. These items can be used to characterize work conditions and workers’ clinical features, but the scores for the complaint items are not included in the total sum.

### Study I: cross-cultural adaptation of the MUEQ to Brazilian Portuguese

**Figure 1:**
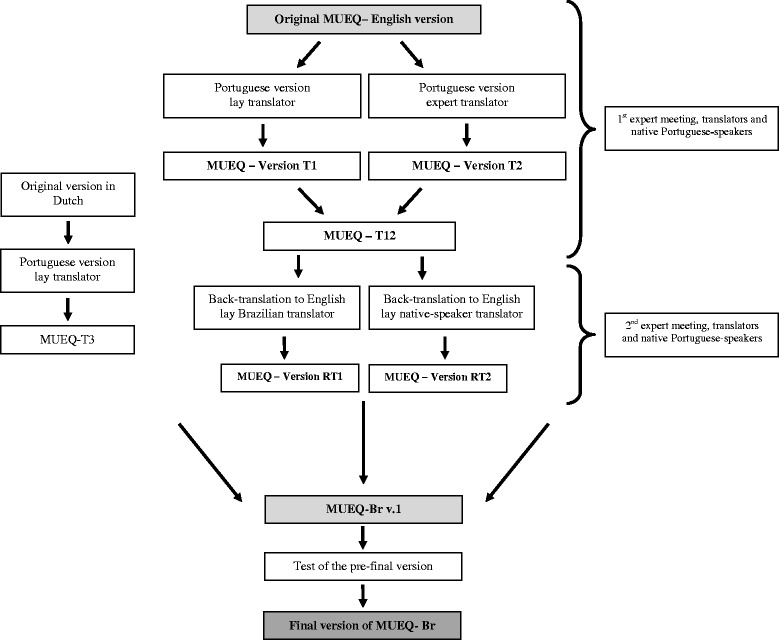
**Flowchart of the process of cross cultural adaptation to Brazilian Portuguese of the Maastricht Upper Extremity Questionnaire.**

Before initiating this study, we obtained written permission from the author of the original MUEQ, Prof. Shahla Eltayeb. The process was performed in three phases: English-to-Portuguese translation, synthesis of the translation (committee translation agreement), back-translation, a consensus committee for the pre-final version, and a field test of the pre-final version as recommended by Beaton et al. [23] (see Figure 1).

Once the English version was obtained from the author [2], it was translated into Portuguese. This version was translated into Brazilian Portuguese by two translators who were fluent in both languages and whose native language was Portuguese; one was a lay translator and the other was an expert translator. Both Brazilian Portuguese versions were compared and synthesized by a committee of translators that included 11 members who were researchers, experts on ergonomics, and civilians [23]. This process resulted in the synthesized version T12 (see Figure 1).

Following this phase, two new translators (one native English-speaker and one Brazilian fluent in English) were invited to translate the T12 version into English (i.e., back-translation; see Figure 1). These two translators did not have experience in the health field and were “blind” to all the previous versions of the questionnaire [23]. Beyond the English to Portuguese translation, a Dutch to Portuguese translation was also performed. One lay translator, whose native language was Brazilian Portuguese, who was fluent in Dutch had previously translated the original MUEQ into Dutch (see Figure 1). Only back translation from Portuguese (T12) to Dutch (T3) was performed because it was not possible to find another Brazilian native who was fluent in Dutch to perform the initial translation.

In following phase, the same committee as above met again (see Figure 1). The committee’s role was to create the pre-final version that would be administered in the field tests [24].

### Test of the pre-final version

The test of the pre-final version consisted of administering the questionnaire to a sample of participants that corresponded to the target population. The purpose of this step was to assess the acceptability of the tool [25].

The pre-final version of the questionnaire was first administered to 15 subjects, and the index of doubts and suggestions obtained was lower than (0.07%). Thus, it was not necessary to reformulate the questionnaire [23]. However, suggestions that did not change the meaning of the questions were incorporated. The sample size in this phase was 55 workers, which is a good sample size as suggested by the COSMIN protocol [26].

The acceptability of the tool was controlled by means of an open-field form so that the workers who answered the pre-final version of the MUEQ-Br could report any doubts, their impressions and incomprehension of each item, answer choices, item headings, instructions, and the tool’s layout.

### Study II: reproducibility of the tool

The volunteers who completed the final version of the MUEQ-Br (Additional file 1) were invited to complete the questionnaire again one week later in order to verify answer reproducibility. The volunteers were asked whether there had been any changes to their work conditions and musculoskeletal complaints, and they were excluded from the reproducibility study if such changes had occurred. The MUEQ was self-administered.

### Study III: validation

The internal consistency and confirmatory factor analysis of the instrument were assessed. Internal consistency is the degree to which the items in the questionnaire are correlated [24,26].

Factor analysis is an important statistical phase for the validation of questionnaires. Its objective can be to reduce data, assess the structural factors (i.e., the dimensions of the tool), or investigate whether the questionnaire shows the same dimensions across different groups [27]. Because the MUEQ factor structure has been previously described [11,18,19], a better approach is to use confirmatory factor analysis to assess structural validity.

The confirmatory factor analysis was applied to confirm the model adopted in previous publications [2,11,18,19]. The sample of workers was the same used to calculate the internal consistency (n = 386) (see Table 1). The sample size used followed the recommendation of the COSMIN manual [20,26], which states that the sample size should be 5–7 times the number of items with a minimum of 100 individuals. Considering that there are 55 questions across the six domains of the MUEQ-Br, a sample based on COSMIN recommendations would need to have at least 385 respondents (see Table 2) to verify the consistency and confirmatory factor analysis of the MUEQ-Br, and the current study had 386.

**Table 2:** **Item total correlation, Cronbach’s α for excluded items, and mean Cronbach’s α for each domain of the Brazilian Portuguese version of the Upper Extremity Questionnaire revised (MUEQ-Br revised)**

**Question**	**Item total correlation**	**Cronbach’s α for excluded items**
**Work Station** (Maximum domain score = 6 points)		
1. My desk (table) at work has suitable height.	0.52	0.71
2. I can adjust my chair height.	0.28	0.70
3. The chair I use during my work supports my lower back.	0.35	0.70
4. My keyboard is placed directly in front of me.	0.36	0.72
5. The screen is placed directly in front of me.	0.48	0.70
6. I have enough space to work at my office.	0.36	0.70
**Mean Cronbach’s α for the domain = 0.70**		
**Body posture** (Maximum domain score = 18 points)		
7. During my work, I sit in an awkward body posture.	0.51	0.73
8. At work, I perform repetitive tasks.	0.59	0.72
9. I find my job physically exhausting.	0.65	0.72
10. My head is twisted towards the left or right.	0.42	0.74
11. My trunk is twisted towards the left or right.	0.68	0.71
12. My trunk is in a misaligned position	0.44	0.74
**Mean Cronbach’s α for the domain = 0.75**		
**Job control** (Maximum domain score = 27 points)		
13. I decide how to perform my job task.	0.54	0.74
14. I participate with others in decision making.	0.61	0.72
15. I decide my own task changes.	0.60	0.73
16. I determine the time and speed of job tasks.	0.53	0.74
17. I solve work problems by myself.	0.38	0.74
18. My work develops my abilities.	0.68	0.72
19. In my work, I learn new things.	0.64	0.73
20. I have to be creative in my work.	0.62	0.72
21. I undertake different tasks in my work.	0.47	0.74
**Mean Cronbach’s α for the domain = 0.75**		
**Job demand** (Maximum domain score = 21 points)		
22. I work under extensive pressure.	0.66	0.74
23. I find it difficult to finish my work tasks on time.	0.71	0.73
24. I take extra hours to finish my work tasks.	0.61	0.74
25. I do not have enough time to finish my job task.	0.63	0.74
26. At work, I speed to finish my tasks on time.	0.65	0.75
27. I find my work tasks difficult.	0.55	0.76
28. I have too many job tasks.	0.55	0.75
**Mean Cronbach’s α for the domain = 0.77**		
**Break time** (Maximum domain score = 18 points)		
29. I can plan my work breaks.	0.69	0.73
30. I can divide my work time.	0.65	0.73
31. I can decide when to take a break.	0.67	0.74
32. I alternate my body posture.	0.51	0.75
33. I alternate my job task.		0.56
34. I find my work breaks sufficient.	0.57	0.73
**Mean Cronbach’s α for the domain = 0.77**		
**Social Support (**Maximum domain score = 18 points)		
35. The workflow goes smoothly.	0.60	0.75
36. My work task depends on other colleagues.	0.64	0.75
37. My work atmosphere is comfortable.	0.60	0.75
38. If I make a mistake in my work task, I find support from my colleagues.	0.66	0.75
39. If I make a mistake in my work task, I find support from my supervisors.	0.75	0.74
40. My colleagues are friendly.	0.58	0.76
41. My supervisors are friendly.	0.73	0.74
**Mean Cronbach’s α for the domain = 0.77**		

### Statistical analysis

The statistical analysis for reproducibility was performed using intraclass correlation coefficient (ICC), with levels of classification as described by Fleiss et al. [26] and in which ICC <0.40 is considered weak, 0.40 < ICC < 0.7 is considered moderate, and ICC >0.75 is considered excellent. The statistical model specified was a two-way mixed effects model, based on absolute agreement measures [28].

Internal consistency was analyzed using Cronbach’s α coefficient, with results between 0.7 and 0.95 [24], and an item-total correlation between 0.2 and 0.7 was considered acceptable [29]. The Spearman correlation between the standard error of measurement (SEM) of the total score of the questionnaire on test-retest and the time taken(minutes) to complete the questionnaire was calculated. The SEM was calculated using the formula: SEM = SD √1 - ICC, where SD = the standard deviation between scores from the test and retest [30]. For comparisons between mean values among the groups, one-way ANOVAs were applied.

Confirmatory factor analysis was used to test the factor structure of the MUEQ-Br in computer workers. The IBM SPSS AMOS (version 22) was used for conducting the confirmatory factor analysis. Maximum likelihood was used to assess the fit of three different models. The goodness-of-fit for each factor structure was evaluated using several descriptive criteria: (1) consistent Akaike information criterion (CAIC), (2) root mean square error of approximation (RMSEA), (3) the comparative fit index (CFI), (4) the goodness-of-fit index (GFI), (5) the non-normed fit index (NNFI) or Tucker Lewis index (TLI), (6) the expected cross-validation index (ECVI), and (7) chi-square (also called the discrepancy function, likelihood ratio chi-square, or chi-square goodness of fit) (CMIN). The CAIC is a goodness-of-fit measure that adjusts the model’s chi-square to penalize for model complexity and sample size [31]. Low measures indicate better fit. The RMSEA quantifies the divergence between the data and the proposed model per degree of freedom. Values below 0.08 indicate an adequate fit [31]. The CFI, GFI, and NNFI measure how much better the model fits as compared to a baseline model in which the observed items are assumed to be uncorrelated. These indices are relatively independent of sample size. The CFI avoids underestimation of fit in small sample sizes. For the CFI and GFI, values above 0.90 indicate an adequate fit, and values above 0.95 indicate a good to very good fit. The ECVI is a relative measure to compare competing models: The model with the lowest value has the best fit [31]. The CMIN/DF (degrees of freedom) should be less than three [32]. The magnitudes of factor loadings for each variable were considered when analyzing the items’ contributions to the model. Variables with a factor loading of 0.4 or greater [33] were considered representative of the construct being measured in each domain.

All analyses were performed using the Statistical Package for the Social Sciences (SPSS) for Windows and IBM SPSS AMOS, version 22 (IBM, SPSS Inc., Chicago, USA).

## Results

### Cross-cultural adaptation, pre-final test, and reproducibility of the MUEQ-Br

During the translation and back-translation processes, only some minor cultural-linguistic adaptations were performed that did not change the content of the MUEQ items. The consensus group agreed that the MUEQ-Br demonstrated semantic and grammatical equivalence.

On the pre-final version, the questions that raised doubts were: (1) “There is unwanted air in the office”, (2) “For more than two hours per day, I sit with lifted shoulders”, (3) “When I work, my head is bent”, (4) “My head is twisted towards the left or right” (work-station domain), (5) “My trunk is twisted towards the left or right” (work-station domain), and (6) “I have too many job tasks” (job-demand domain). The prevalence index of doubts or suggestions by respondents on the pre-final-test version was very low (from 0.02 to 0.07). Thus, it was not necessary to reformulate the questionnaire, and the suggestions that did not change the meaning of the questions were included.

The reproducibility of the questionnaire was tested, and the agreement level was verified and considered excellent (ICC >0.75) for every domain and the total score of the questionnaire (see Table 3). There was no correlation between the duration to complete the questionnaire and error between the test-retest scores (Spearman Ro = 0.072, p = 0.61).

**Table 3:** **Mean intraclass correlation coefficient (ICC) values and 95% confidence intervals for the reproducibility of the scores of the MUEQ-Br domains**

**Domain**	**ICC (95%)**
Work station	0.94 (0.90–0.96)
Body posture	0.85 (0.74–0.91)
Job control	0.84 (0.71–0.90)
Demand	0.95 (0.91–0.97)
Break time	0.94 (0.89–0.96)
Social support	0.87 (0.77–0.92)
Complaints	0.98 (0.96–0.98)
**Total**	0.95 (0.90–0.97)

There was a difference between the mean ages of the participants in the different phases of the study (for the pre-test-final-version group, n = 55, and for the validation-study group, n = 386, F = 4.5, p = 0.01). Despite the statistical difference that was within the 95% confidence interval, all the groups were in the third decade of life and did not have physiological implications. Moreover, there was no difference between groups in terms of the number of years of computer use at work (10 to 13 years).

### Internal consistency and confirmatory factor analysis of the MUEQ-Br

Cronbach’s alpha for each domain was greater than 0.70 [24], and the item-total correlation for each domain was between 0.28 and 0.75, which is inside the acceptable range (0.2–0.7) [29] (see Table 2).

The model with seven (Model 1: 59 questions) and six (Model 2: 50 questions) factors was tested, and the results are described in Table 4. Previously, three models were tested. The current models were chosen because they were used in previously published versions of the MUEQ and because biopsychosocial aspects are covered by the domains previously described (biological or physical aspects: work-station, body-posture, and break-time domains; psychosocial aspects: job-control, job-demand, break-time, and social-support domains). The work-environment domain was related more to the workplace’s physical aspects than to the worker’s physiological aspects. For this reason, that domain was eliminated from the model structure tested. The model from Bekiari et al. [18] with seven domains (including work environment with nine questions) and the model from Eltayeb et al. [2] with six domains (excluding work environment) were used. However, for these two models, the results showed an inadequate fit; CFI, GFI, and NNFI were below 0.90, and there were greater values of CAIC and ECVI (see Table 4).

**Table 4:** **Goodness-of-fit indices for several MUEQ-Br revised factor solutions obtained by confirmatory factor analysis (N = 386)**

	***X*** ^**2**^ **(df)**	**CAIC** *	**CFI** ^**£**^	**GFI** ^**ψ**^	**NNFI** ***	**ECVI& (90% CI)**	**RMSEA** ** **(90% CI)**
Model 1	3,775.47 (1524)	4,672.78	0.66	0.72	0.64	10.47 (10.01–10.96)	0.06 (0.059–0.066)
Model 2	1,962.45 (990)	3,256.24	0.83	0.75	0.82	6.06 (5.74–6.40)	0.05 (0.041–0.056)
Model 3	1,145.28 (705)	2,230.40	0.91	0.90	0.90	3.78 (3.55–4.04)	0.04 (0.036–0.044)

*Note.* Model 1 had seven domains and 59 questions and was based on Bekiari et al. [11]. Model 2 had six domains (excluding work environment) and 50 questions and was based on Eltayeb et al. [2]. Model 3 had six domains (excluding work environment) and 41 questions (nine questions excluded: one from work station, five from body posture, two from break time, and one from social support).

*CAIC: consistent with the Akaike information criterion.

**RMSEA: root mean square error of approximation; “90% CI” = 90% confidence interval for RMSEA.

***NNFI: non-normed fit index.

^£^CFI: comparative fit index.

^ψ^GFI: goodness-of-fit index.

&ECVI: expected cross-validation index and 90% CI.

Considering the regression results (maximum likelihood) for Model 2 (50 questions), an additional nine questions were not significant in the final model analysis: one question from the work-station domain (“The chair I use during work supports my lower back”), five questions from the body-posture domain (“During my work, I keep a good work posture”, “At work, I sit for long hours in one position”, “For more than two hours per day, I sit with lifted shoulders”, “When I key, my hand is placed in a straight line with my lower arm”, and “When I work, my head is bent”), two questions from the break-time domain (“I alternate in my body posture” and “I find my work breaks sufficient”), and one question from the social-support domain (“My work tasks depend on other colleagues”). When Model 3 was tested (i.e., without those nine questions), the fit achieved adequate or better-than-adequate values as described in Table 4. The CFI, GFI, and NNFI achieved 0.90, and the CAIC, chi-square, and ECIV demonstrated better values. The RMSEA for Model 3 was the lowest observed (see Table 4).

**Figure 2:**
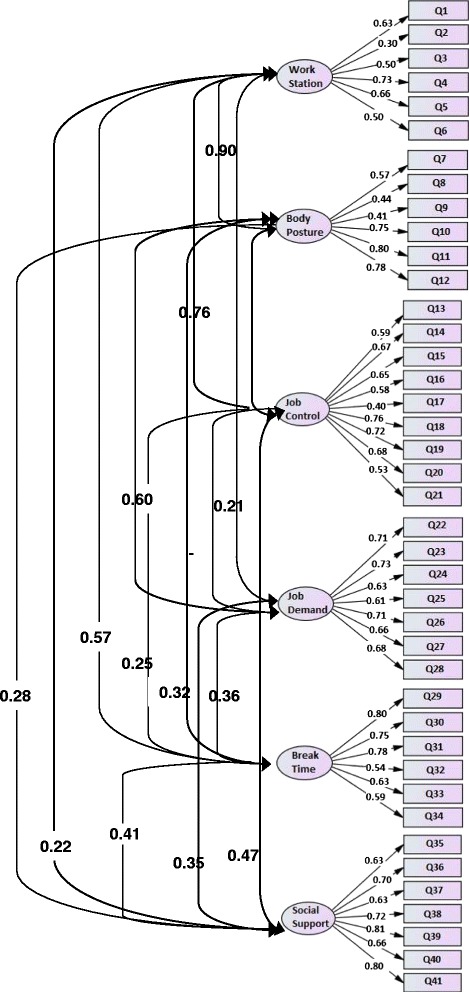
**Confirmatory Factor Analysis of the Brazilian Portuguese of the revised Maastricht Upper Extremity Questionnaire (MUEQ-Br revised).** Factor loadings of each domain item and correlations.

Figure 2 demonstrates the better model fit that was obtained (six domains and 41 questions, see Additional file 2) and the factor loadings obtained for each question. Values were greater than 0.4 for the majority of the items for each domain, as recommended in the literature [32], except for question 2 in the work-station domain (“I can adjust my chair height”), which obtained a factor load of 0.3. However, it was kept in the model because it was significant in this domain according to the regression analysis (see Figure 2). The new score of each domain of the 41-item Brazilian MUEQ-Br revised is shown in Table 2.

## Discussion

The objective of this study was to perform a cross-cultural adaptation of the MUEQ to Brazilian Portuguese and to verify the psychometric properties of the translated tool (reproducibility, structural validity, and internal consistency) in a sample of Brazilian computer-office workers. The recommendations suggested by the COSMIN manual [20,26] were followed in the cross-cultural adaptation process. The COSMIN [20] recommends testing the reproducibility, internal consistency, structural validity (within-construct validity) for health assessment tools. The confirmatory factor analysis confirmed the pre-specified factor structure of the model that has been reported by Eltayeb et al. [2] with six domains: work station, body posture, job control, job demand, social support, and break time (excluding nine questions).

The reproducibility of the MUEQ-Br tool and the subscales showed adequate values and sample size [20]. None of the previously published versions of the MUEQ [2,11,18,19] assessed the reproducibility (test-retest) of the tool.

The internal consistency of the factors was confirmed by measuring Cronbach’s alpha. Cronbach’s alpha coefficient of the MUEQ-Br revised was greater than 0.70 for all the subscales. This value is higher than that for the other versions; Cronbach’s alpha was between 0.52 and 0.89 for the Greek version [18], 0.54 and 0.85 for the Dutch version [2], and 0.48 and 0.94 for the Arabic version [11].

In this study, three models were tested to verify the factor structure of the MUEQ-Br revised. The model from Bekiari et al. [18] with seven domains (including nine work-environment questions) and the model from Eltayeb et al. [2] with six domains (excluding the work-environment domain) showed inadequate fit and index values (CFI, GFI, and NNFI were below 0.90, and there were greater values for CAIC and ECVI).

Considering the results of the confirmatory factor analysis of Eltayeb et al.’s [2] model, a total of nine questions were not significant in the current model analyzed. However, only the reduced model from Eltayeb et al. [2] achieved adequate index-fit values (CFI, GFI, and NNFI greater than 0.90) and better values of CAIC, chi-square, and ECIV. Because 18 questions were excluded from the original 59-item MUEQ, we suggest that the 41-item Brazilian Portuguese version be considered a revised version.

The question assessing whether the worker’s chair supports the lower back was not significant in the model, most likely because the majority of the chairs available in workplaces are actually designed to accommodate the lower back. The question, “At work, I sit for long hours in one position” describes a situation that is uncommon for many office-work situations because administrative workers generally alternate between tasks that involve a sitting position and tasks that involve changes in posture (e.g., finding a document, administrative meetings, etc.). One hypothesis is that excluding four questions related to body posture (“For more than two hours per day, I sit with lifted shoulders,” “When I key, my hand is placed in a straight line with my lower arm”, “When I work, my head is bent”, and “I alternate my body posture”) could increase the level of applicability of the questionnaire. Moreover, two questions (“I find my work breaks sufficient” and “My work tasks depend on other colleagues”) may not be representative of the office-work environment because work breaks and dependence on colleagues are more common in production-line work.

The MUEQ-Br revised has a fewer number of questions and demonstrated good psychometric property indexes. The results of the exploratory factor analysis of the original MUEQ [2] were previously verified, and approximately 50% of the variance for each scale could be explained, which can be considered an acceptable index [34]. However, for two domains (work station and body posture), the explained variance was lower than the recommended values (44.4% and 43.9%, respectively), suggesting that the factor structures previously reported may not be an ideal model to represent the construct as assessed by the MUEQ. Curiously, six of nine questions that were excluded from the final model of the MUEQ-Br revised were from these two domains.

There are few tools available in Brazilian Portuguese to assess worker health that have been cross-culturally adapted. The Quick Exposure Check, which evaluates general ergonomic risk in workers, was adapted to Brazilian Portuguese and the validation phases have been conducted [15]. In addition, the Job Factors Questionnaire, which is a generic tool to asses work factors that may be related to the development of musculoskeletal complaints [16], and the Nordic Musculoskeletal Questionnaire, which aims to identify the main musculoskeletal-disorder symptoms in general workers [17], are available. However, the psychometric properties of the Quick Exposure Check and the Job Factors Questionnaire have not been adequately tested, and neither tool’s exploratory or confirmatory factor analyses have been verified. Despite the existence of these tools, the MUEQ is the only specific tool for assessing the biopsychosocial reports of computer-office workers. The innovative character of this study is that it provides a tool in Brazilian Portuguese that can increase understanding of the major musculoskeletal complaints that affect these workers as well as the relationship with ergonomic and psychosocial factors, thus facilitating the conduction of broad spectrum studies.

We did not find a correlation between the standard error of measurement (SEM) from the MUEQ-Br revised test-retest scores and the duration to complete the questionnaire. We suggest that when the workers answered each question, they did so accurately, and the high reproducibility indexes confirm this suggestion.

One of the limitations of this work is the low response rate of 62%; of the 627 volunteers approached, only 386 choose to complete the MUEQ-Br revised. Eltayeb et al. [2] reported a response rate of 44% when the MUEQ was administered in the Netherlands, and the literature considers 60% an acceptable response rate for surveys [35]. For this reason, our results must be considered in light of this limitation.

One could argue that the low response rate suggests a decrease in the representativeness of the results. However, the most important aspect is the education level of the Brazilian population. Nevertheless, the majority of office workers in Brazil must have at least a high school degree or technician formation in order to work, and they need to receive at least 11 years of formal education. Because the education-level requirement is country-wide, it is likely that computer-office workers from different parts of Brazil will understand the concepts of the MUEQ-Br revised.

Some aspects that may explain the low response rate are the length of the questionnaire (which will be minimized because the Portuguese version is shorter) and the workers’ fear of reprisals (the worker was invited to complete the questionnaire in their work environments). This latter issue could discourage volunteer participation and is a common problem for workers in the health area [36]. Another aspect that has been identified in the literature is requiring a signature before the start of an epidemiologic survey; this may reduce the response rate [37] and is a compulsory practice in research in Brazil. In future prevalence studies, we suggest that the questionnaire be tested on different samples from different parts of Brazil in order to improve the representativeness of the results.

## Conclusions

In conclusion, the MUEQ-Br revised presented satisfactory measurement properties according to cross-cultural validity, reproducibility, internal consistency, and factor analysis. The results provide a basis for using the 41-item MUEQ-Br revised for the assessment of Brazilian computer-office workers’ perception about psychosocial and ergonomic aspects and musculoskeletal complaint characterization.

## Additional files

Additional file 1:
**Maastricht Upper Extremity Questionnaire English version.**
Additional file 2:
**Final Brazilian Portuguese Version of the Revised Maastricht Upper Extremity Questionnaire (MUEQ-Br revised).**
