# Supplementary material for: The Brazilian Portuguese version of the revised Maastricht Upper Extremity Questionnaire (MUEQ-Br revised): translation, cross-cultural adaptation, reliability, and structural validation
Source: BMC Musculoskelet Disord. 2015 Feb 25;16:41. doi: 10.1186/s12891-015-0497-2 (PMC4352257; doi:10.1186/s12891-015-0497-2)
Supplement: Additional file 1: — Maastricht Upper Extremity Questionnaire English version. [file 12891_2015_497_MOESM1_ESM.docx]

# APPENDIX 1 –Maastricht Upper Extremity Questionnaire English version

**General Information:**

|  | Gender | □ Male □ Female |
| --- | --- | --- |
|  | Surname | ____________________________________ |
|  | Date of birth? | ___-___-19___ |
|  | Where do you work? | □ Heerlen  □ Maastricht  □ Both |
|  | What is your current position? | ____________________________________ |
|  | How long have you been working in this position? | ___ Year |
|  | How many days do you work per week?  (*over time not included*) | ___ Day |
|  | How many hours do you work per day?  *(breaks and over time not included)* | ___ Hour |
|  | How many hours per working day do you work behind your computer? | ___ Hour |

**Work Station**

|  | My desk (table) at work has suitable height. | □ No  □ Yes |
| --- | --- | --- |
|  | I can adjust my chair height. | □ No  □ Yes |
|  | When I use the mouse device, my arm is supported by the table. | □ No  □ Yes |
|  | The chair I use during work supports my lower back. | □ No  □ Yes |
|  | My keyboard is placed directly in front of me. | □ No  □ Yes |
|  | The screen is placed directly in front of me. | □ No  □ Yes |
|  | I have enough space to work at my office. | □ No  □ Yes |

**Body Posture**

|  | |  | Always | Often | Sometimes | Seldom | Never |
| --- | --- | --- | --- | --- | --- | --- | --- |
|  | | During my work I keep a good work posture. | □ | □ | □ | □ | □ |
|  | | At work I sit for long hours in one position. | □ | □ | □ | □ | □ |
|  | | For more than two hours per day I sit with lifted shoulders. | □ | □ | □ | □ | □ |
|  | | During my work I sit in awkward posture. | □ | □ | □ | □ | □ |
|  | In work I perform repetitive tasks. | □ | □ | □ | □ | □ |  |
|  | I find my job physically exhausting. | □ | □ | □ | □ | □ |  |
|  | When I key my hand is placed in a straight line with my lower arm. | □ | □ | □ | □ | □ |  |
|  | When I work my head is bended. | □ | □ | □ | □ | □ |  |
|  | Head is twisted towards the left or right. | □ | □ | □ | □ | □ |  |
|  | Trunk is twisted towards the left or right. | □ | □ | □ | □ | □ |  |
|  | My Trunk is in asymmetrical position. | □ | □ | □ | □ | □ |  |

**Job Control**

|  |  | Always | Often | Sometimes | Seldom | Never |
| --- | --- | --- | --- | --- | --- | --- |
|  | I decide how to perform my job task. | □ | □ | □ | □ | □ |
|  | I participate with others in decision taking. | □ | □ | □ | □ | □ |
|  | I decide my own task changes. | □ | □ | □ | □ | □ |
|  | I determine the time & speed job tasks. | □ | □ | □ | □ | □ |
|  | I solve work problems by my self. | □ | □ | □ | □ | □ |
|  | My work develops my abilities. | □ | □ | □ | □ | □ |
|  | In my work I learn new things. | □ | □ | □ | □ | □ |
|  | I have to be creative in my work. | □ | □ | □ | □ | □ |
|  | I under take different tasks in my work. | □ | □ | □ | □ | □ |

**Job Demand**

|  |  | Always | Often | Sometimes | Seldom | Never |
| --- | --- | --- | --- | --- | --- | --- |
|  | I work under extensive work pressure. | □ | □ | □ | □ | □ |
|  | I find it difficult to finish my tasks on time. | □ | □ | □ | □ | □ |
|  | I take extra hours to finish my job tasks. | □ | □ | □ | □ | □ |
|  | I have no enough time to finish my job task. | □ | □ | □ | □ | □ |
|  | At work I speed to finish my tasks on time. | □ | □ | □ | □ | □ |
|  | I find my work tasks difficult. | □ | □ | □ | □ | □ |
|  | I have too many job tasks. | □ | □ | □ | □ | □ |

**Break Time**

|  |  | Always | Often | Sometimes | Seldom | Never |
| --- | --- | --- | --- | --- | --- | --- |
|  | I can plan my work breaks. | □ | □ | □ | □ | □ |
|  | I can divide my work time. | □ | □ | □ | □ | □ |
|  | I can decide when to take a break. | □ | □ | □ | □ | □ |
|  | I alternate in my body posture. | □ | □ | □ | □ | □ |
|  | I alternate in my job task. | □ | □ | □ | □ | □ |
|  | I perform job task without computer. | □ | □ | □ | □ | □ |
|  | After two hours I take a break for 10 minutes. | □ | □ | □ | □ | □ |
|  | I find my work breaks sufficient. | □ | □ | □ | □ | □ |

**Work environment**

|  |  | Always | Often | Sometimes | Seldom | Never |
| --- | --- | --- | --- | --- | --- | --- |
|  | I find my work environment good. | □ | □ | □ | □ | □ |
|  | The air inside the office is too dry. | □ | □ | □ | □ | □ |
|  | The air inside the office is too cold. | □ | □ | □ | □ | □ |
|  | In the office there is unwanted air. | □ | □ | □ | □ | □ |
|  | There is available fresh air in my work. | □ | □ | □ | □ | □ |
|  | My work environment is noisy. | □ | □ | □ | □ | □ |
|  | My work place is too bright. | □ | □ | □ | □ | □ |
|  | I gaze at the computer screen. | □ | □ | □ | □ | □ |
|  | The computer screen reflects the office lights. | □ | □ | □ | □ | □ |

**Social Support**

|  |  | Always | Often | Sometimes | Seldom | Never |
| --- | --- | --- | --- | --- | --- | --- |
|  | The work flow goes smoothly. | □ | □ | □ | □ | □ |
|  | I can ask and enquire in my work. | □ | □ | □ | □ | □ |
|  | My work tasks depend on other colleges. | □ | □ | □ | □ | □ |
|  | My work atmosphere is comfortable. | □ | □ | □ | □ | □ |
|  | If I made a mistake in my work task I find support from my colleges. | □ | □ | □ | □ | □ |
|  | If I made a mistake in my work task I find support from my supervisors. | □ | □ | □ | □ | □ |
|  | My colleagues are friendly. | □ | □ | □ | □ | □ |
|  | My supervisors are friendly. | □ | □ | □ | □ | □ |

**Complaints**

During the past year I had pain or complaints for at least one week in one or more of the following body regions

|  | Neck | □ No  □ Yes |  |  |
| --- | --- | --- | --- | --- |
|  | Shoulder(s) | □ No  □ Yes → | If Yes, | □ Left  □ Right  □ Both |
|  | Upper Arm | □ No  □ Yes → | If Yes, | □ Left  □ Right  □ Both |
|  | Elbow (s) | □ No  □ Yes → | If Yes, | □ Left  □ Right  □ Both |
|  | Lower Arm | □ No  □ Yes → | If Yes, | □ Left  □ Right  □ Both |
|  | Wrists | □ No  □ Yes → | If Yes, | □ Left  □ Right  □ Both |
|  | Hand | □ No  □ Yes → | If Yes, | □ Left  □ Right  □ Both |

**From here (upper musculoskeletal extremity) would be used to represent (neck, shoulder, hand, wrist, arm and elbow)**

|  | During the past year I had pain/complaint/disability in my upper musculoskeletal extremity | □ No  □ Yes |
| --- | --- | --- |
|  | The longest period of complaint (in the past year) whereby I could not perform my daily activity was | □ ____Days  □ ____weeks |
|  | During the past year I was referred to the physician due to my upper extremity pain? | □ No  □ Yes→ The physician. Diagnosis of the complaint was? _________ |
|  | What kind of treatment did you receive (during the past year) | □ Physiotherapy  □ Medication  □ Operation  □ Other ______________ |
|  | Because of my upper extremity pain I have lost a job before | □ No  □ Yes |
|  | Because of my upper extremity complaints (during the past year) I was absent from work | □ No  □ Yes |
|  | Due to upper extremity complaints in the past year my activities were hindered  - in my work  - in my leisure time | □ No  □ Yes  □ No  □ Yes |
|  | My complaints are due to a previous accident. | □ No  □ Yes |

**The next questions are related to pain complaints in the neck, shoulder, hand, wrist, and elbow in the past year**

|  | I feel pain in my upper musculoskeletal extremity as soon as I finish work | □ No  □ Yes → | This pain disappears after a short rest | □ No  □ Yes |
| --- | --- | --- | --- | --- |
|  | I feel fatigue and exhaustion in my upper musculoskeletal extremity | □ No  □ Yes → | This complaint disappears after a short rest | □ No  □ Yes |
|  | I feel stiffness in my finger | □ No  □ Yes → | This stiffness disappears after a short rest | □ No  □ Yes |
|  | I feel numbness in my fingers | □ No  □ Yes → | This numbness continues after a short rest | □ No  □ Yes |
|  | I feel tingling in my fingers | □ No  □ Yes → | This tingling continue after work | □ No  □ Yes |
|  | I feel weakness in my upper musculoskeletal extremity | □ No  □ Yes → | This weakness continue after work | □ No  □ Yes |
|  | I suffer from swelling in my hands | □ No  □ Yes → | This swelling continue after work | □ No  □ Yes |
|  | I feel swelling/ stiffness in my upper musculoskeletal extremity | □ No  □ Yes |  |  |
|  | I feel continuous pain in my upper musculoskeletal extremity | □ No  □ Yes |  |  |
|  | I feel a change in the colour, temperature, sweating in my upper musculoskeletal extremity | □ No  □ Yes |  |  |
|  | I use mouse pad, file holder, foot supporter to reduce upper musculoskeletal extremity pain | □ No  □ Yes |  |  |
|  | I use neck collar or belts or other to reduce upper musculoskeletal extremity pain | □ No  □ Yes |  |  |
