# Supplementary material for: The Brazilian Portuguese version of the revised Maastricht Upper Extremity Questionnaire (MUEQ-Br revised): translation, cross-cultural adaptation, reliability, and structural validation
Source: BMC Musculoskelet Disord. 2015 Feb 25;16:41. doi: 10.1186/s12891-015-0497-2 (PMC4352257; doi:10.1186/s12891-015-0497-2)
Supplement: Additional file 2: — Final Brazilian Portuguese Version of the Revised Maastricht Upper Extremity Questionnaire (MUEQ-Br revised). [file 12891_2015_497_MOESM2_ESM.docx]

| APPENDIX 2 - Final Brazilian Portuguese Version of the Revised Maastricht Upper Extremity Questionnaire (MUEQ-Br revised) **Informações Gerais** | | | | | | | | | | | | | | | | | | | | | | | | | | | | | | | | | | | | | |  |
| --- | --- | --- | --- | --- | --- | --- | --- | --- | --- | --- | --- | --- | --- | --- | --- | --- | --- | --- | --- | --- | --- | --- | --- | --- | --- | --- | --- | --- | --- | --- | --- | --- | --- | --- | --- | --- | --- | --- |
| Gênero | | | | | □ Masculino □ Feminino | | | | | | | | | | |  | | | | | | | | | | | | |  | | | | | | | | |  |
| Sobrenome | | | | | _______________________________________ | | | | | | | | | | | | | | | | | | | | | | | | | | | | | | | | |  |
| Data de nascimento | | | | | ____-____-______ | | | | | | | | | | |  | | | | | | | | | | | | | | | | | | | | | |  |
| Onde você trabalha? | | | | | Cidade/Estado/País _____________________________ | | | | | | | | | | | | | | | | | | | | | | | | | | | | | | | | |  |
| Qual seu cargo atual? | | | | | _______________________________________ | | | | | | | | | | | | | | | | | | | | | | | | | | | | | | | | |  |
| Há quanto tempo você trabalha nesse cargo? ____ Ano(s)  Há quanto tempo você trabalha utilizando computador? (considere empregos anteriores) ____ Ano(s) | | | | | | | | | | | | | | | | | | | | | | | | | | | | | | | | | | | | | |  |
| Quantos dias você trabalha por semana (Não incluindo hora extra) | | | | | | | | | | | | | | | | | | ____ Dia(s) | | | | | | | | | | |  | | | | | | | | |  |
| Quantas horas você trabalha por dia? (Não incluindo intervalos e hora extra) | | | | | | | | | | | | | | | | | | | | | | ____ Hora(s) | | | | | | |  | | | | | | | | |  |
| Quantas horas por dia de trabalho você trabalha usando um computador? | | | | | | | | | | | | | | | | | | | | | | ____ Hora(s) | | | | | | |  | | | | | | | | |  |
|  | | | | | | | | | | | | | | | | | | | | | | | | | | | | | | | | | | | | | |  |
| **Posto de trabalho** | | | | | | | | | | | | | | | |  | | | | | | | | | | | | |  | | | | | | | | |  |
| 1. Minha mesa de trabalho tem altura adequada. | | | | | | | | | | | | | | | | | | | | □ Não | | | | | | | | | □ Sim | | | | | | | | |  |
| 2. Eu posso ajustar a altura da minha cadeira. | | | | | | | | | | | | | | | | | | | | □ Não | | | | | | | | | □ Sim | | | | | | | | |  |
| 3. Quando eu uso o mouse, meu braço fica apoiado sobre a mesa. | | | | | | | | | | | | | | | | | | | | □ Não | | | | | | | | | □ Sim | | | | | | | | |  |
| 4. O teclado do meu computador fica diretamente em minha frente. | | | | | | | | | | | | | | | | | | | | □ Não | | | | | | | | | □ Sim | | | | | | | | |  |
| 5. A tela do meu computador fica diretamente em minha frente. | | | | | | | | | | | | | | | | | | | | □ Não | | | | | | | | | □ Sim | | | | | | | | |  |
| 6. Eu tenho espaço suficiente para trabalhar em meu escritório. | | | | | | | | | | | | | | | | | | | | □ Não | | | | | | | | | □ Sim | | | | | | | | |  |
|  | | | | | | | | | | | | | | | |  | | | | | | | | | | | | |  | | | | | | | | |  |
| **Postura Corporal** | | | | | | | | | | | | | | | |  | | | | | | | | | | | | |  | | | | | | | | |  |
|  | | | | | | | | | | | | | | | | **Sempre** | | | **Frequentemente** | | | | | | **Às vezes** | | | | | **Raramente** | | | | | **Nunca** | | |  |
| 7. Durante meu trabalho eu sento com uma postura desajeitada. | | | | | | | | | | | | | | | | □ | | | □ | | | | | | □ | | | | | □ | | | | | □ | | |  |
| 8. No meu trabalho eu realizo repetidas tarefas. | | | | | | | | | | | | | | | | □ | | | □ | | | | | | □ | | | | | □ | | | | | □ | | |  |
| 9. Eu acho meu trabalho fisicamente desgastante. | | | | | | | | | | | | | | | | □ | | | □ | | | | | | □ | | | | | □ | | | | | □ | | |  |
| 10. Minha cabeça fica virada para a esquerda ou direita durante o trabalho. | | | | | | | | | | | | | | | | □ | | | □ | | | | | | □ | | | | | □ | | | | | □ | | |  |
| 11. Meu tronco fica virado para a esquerda ou direita durante o trabalho. | | | | | | | | | | | | | | | | □ | | | □ | | | | | | □ | | | | | □ | | | | | □ | | |  |
| 12. Meu tronco fica em uma posição desalinhada. | | | | | | | | | | | | | | | | □ | | | □ | | | | | | □ | | | | | □ | | | | | □ | | |  |
| **Controle do Trabalho** | | | | | | | | | | | | | | | |  | | |  | | | | | |  | | | | |  | | | | |  | | |  |
|  | | | | | | | | | | | | | | | | **Sempre** | | | **Frequentemente** | | | | | | **Às vezes** | | | | | **Raramente** | | | | | **Nunca** | | |  |
| 13. Eu decido como realizar minhas tarefas de trabalho. | | | | | | | | | | | | | | | | □ | | | □ | | | | | | □ | | | | | □ | | | | | □ | | |  |
| 14. Eu participo com os outros na tomada de decisões. | | | | | | | | | | | | | | | | □ | | | □ | | | | | | □ | | | | | □ | | | | | □ | | |  |
| 15. Eu decido minha rotina de tarefas. | | | | | | | | | | | | | | | | □ | | | □ | | | | | | □ | | | | | □ | | | | | □ | | |  |
| 16. Eu determino o tempo e a velocidade das tarefas. | | | | | | | | | | | | | | | | □ | | | □ | | | | | | □ | | | | | □ | | | | | □ | | |  |
| 17. Eu resolvo problemas de trabalho sozinho. | | | | | | | | | | | | | | | | □ | | | □ | | | | | | □ | | | | | □ | | | | | □ | | |  |
| 18. Meu trabalho aprimora minhas habilidades. | | | | | | | | | | | | | | | | □ | | | □ | | | | | | □ | | | | | □ | | | | | □ | | |  |
| 19. No trabalho eu aprendo coisas novas. | | | | | | | | | | | | | | | | □ | | | □ | | | | | | □ | | | | | □ | | | | | □ | | |  |
| 20. Eu tenho que ser criativo em meu trabalho. | | | | | | | | | | | | | | | | □ | | | □ | | | | | | □ | | | | | □ | | | | | □ | | |  |
| 21. Eu realizo tarefas variadas em meu trabalho. | | | | | | | | | | | | | | | | □ | | | □ | | | | | | □ | | | | | □ | | | | | □ | | |  |
|  | | | | | | | | | | | | | | | | | | | | | | | | | | | | | | | | | | | | | |  |
| **Demanda de Trabalho** | | | | | | | | | | | | | | | |  | | |  | | | | | |  | | | | |  | | | | |  | | |  |
|  | | | | | | | | | | | | | | | | **Sempre** | | | **Frequentemente** | | | | | | **Às vezes** | | | | | **Raramente** | | | | | **Nunca** | | |  |
| 22. Eu trabalho sob extrema pressão. | | | | | | | | | | | | | | | | □ | | | □ | | | | | | □ | | | | | □ | | | | | □ | | |  |
| 23. Eu encontro dificuldade em terminar minhas tarefas a tempo. | | | | | | | | | | | | | | | | □ | | | □ | | | | | | □ | | | | | □ | | | | | □ | | |  |
| 24. Eu faço hora extra para terminar minhas tarefas. | | | | | | | | | | | | | | | | □ | | | □ | | | | | | □ | | | | | □ | | | | | □ | | |  |
| 25. Eu não tenho tempo suficiente para terminar minha tarefa de trabalho. | | | | | | | | | | | | | | | | □ | | | □ | | | | | | □ | | | | | □ | | | | | □ | | |  |
| 26. No trabalho eu corro para terminar minhas tarefas no prazo. | | | | | | | | | | | | | | | | □ | | | □ | | | | | | □ | | | | | □ | | | | | □ | | |  |
| 27. Eu encontro em meu trabalho tarefas difíceis. | | | | | | | | | | | | | | | | □ | | | □ | | | | | | □ | | | | | □ | | | | | □ | | |  |
| 28. Eu tenho tarefas de trabalho demais | | | | | | | | | | | | | | | | □ | | | □ | | | | | | □ | | | | | □ | | | | | □ | | |  |
|  | | | | | | | | | | | | | | | |  | | |  | | | | | |  | | | | |  | | | | |  | | |  |
| **Pausas** | | | | | | | | | | | | | | | |  | | |  | | | | | |  | | | | |  | | | | |  | | |  |
|  | | | | | | | | | | | | | | | | **Sempre** | | | **Frequentemente** | | | | | | **Às vezes** | | | | | **Raramente** | | | | | **Nunca** | | |  |
| 29. Eu posso planejar minhas pausas do trabalho. | | | | | | | | | | | | | | | | □ | | | □ | | | | | | □ | | | | | □ | | | | | □ | | |  |
| 30. Eu posso dividir meu tempo de trabalho. | | | | | | | | | | | | | | | | □ | | | □ | | | | | | □ | | | | | □ | | | | | □ | | |  |
| 31. Eu posso decidir quando realizar uma pausa. | | | | | | | | | | | | | | | | □ | | | □ | | | | | | □ | | | | | □ | | | | | □ | | |  |
| 32. Eu alterno minha postura corporal. | | | | | | | | | | | | | | | | □ | | | □ | | | | | | □ | | | | | □ | | | | | □ | | |  |
| 33. Eu alterno minhas tarefas de trabalho. | | | | | | | | | | | | | | | | □ | | | □ | | | | | | □ | | | | | □ | | | | | □ | | |  |
| 34. A cada duas horas eu faço uma pausa de dez minutos. | | | | | | | | | | | | | | | | □ | | | □ | | | | | | □ | | | | | □ | | | | | □ | | |  |
|  | | | | | | | | | | | | | | | |  | | |  | | | | | |  | | | | |  | | | | |  | | |  |
|  | | | | | | | | | | | | | | | |  | | |  | | | | | |  | | | | |  | | | | |  | | |  |
| **Suporte Social** | | | | | | | | | | | | | | | |  | | |  | | | | | |  | | | | |  | | | | |  | | |  |
|  | | | | | | | | | | | | | | | | **Sempre** | | | **Frequentemente** | | | | | | **Às vezes** | | | | | **Raramente** | | | | | **Nunca** | | |  |
| 35. O trabalho flui tranquilamente. | | | | | | | | | | | | | | | | □ | | | □ | | | | | | □ | | | | | □ | | | | | □ | | |  |
| 36. Eu posso perguntar e questionar em meu trabalho. | | | | | | | | | | | | | | | | □ | | | □ | | | | | | □ | | | | | □ | | | | | □ | | |  |
| 37. Meu ambiente de trabalho é confortável. | | | | | | | | | | | | | | | | □ | | | □ | | | | | | □ | | | | | □ | | | | | □ | | |  |
| 38. Se eu cometer algum erro nas tarefas de trabalho eu tenho suporte dos meus colegas. | | | | | | | | | | | | | | | | □ | | | □ | | | | | | □ | | | | | □ | | | | | □ | | |  |
| 39. Se eu cometer algum erro nas tarefas de trabalho eu tenho suporte dos meus supervisores. | | | | | | | | | | | | | | | | □ | | | □ | | | | | | □ | | | | | □ | | | | | □ | | |  |
| 40. Meus colegas são amigáveis. | | | | | | | | | | | | | | | | □ | | | □ | | | | | | □ | | | | | □ | | | | | □ | | |  |
| 41. Meus supervisores são amigáveis. | | | | | | | | | | | | | | | | □ | | | □ | | | | | | □ | | | | | □ | | | | | □ | | |  |
|  | | | | | | | | | | | | | | | |  | | |  | | | | | |  | | | | |  | | | |  | | | |  |
| **Queixas** | | | | | | | | | | | | | | | |  | | |  | | | | | |  | | | | |  | | | |  | | | |  |
|  | | | | | | | | | | | | | | | |  | | |  | | | | | |  | | |  | | | | | |  | | | |  |
| **(I)** *Durante os últimos três meses eu tive dor ou queixas, por pelo menos uma semana, em uma ou mais das seguintes regiões corporais:* | | | | | | | | | | | | | | | | | | | | | | | | | | | | | | | | | | | | | |  |
| 45. Pescoço | | | | □ Não | | | | | □ Sim | | | | | | | | | | | | | | | | | | | | | | | | | | | | |  |
| 46. Ombro(s) | | | | □ Não | | | | | □ Sim | | | -> Se sim, | | | | | | | □ Esquerda | | | | | | | □ Direita | | | | | | □ Ambos | | | | | |  |
| 47. Braço(s) | | | | □ Não | | | | | □ Sim | | | -> Se sim, | | | | | | | □ Esquerda | | | | | | | □ Direita | | | | | | □ Ambos | | | | | |  |
| 48. Cotovelo(s) | | | | □ Não | | | | | □ Sim | | | -> Se sim, | | | | | | | □ Esquerda | | | | | | | □ Direita | | | | | | □ Ambos | | | | | |  |
| 49. Antebraço(s) | | | | □ Não | | | | | □ Sim | | | -> Se sim, | | | | | | | □ Esquerda | | | | | | | □ Direita | | | | | | □ Ambos | | | | | |  |
| 50. Punho(s) | | | | □ Não | | | | | □ Sim | | | -> Se sim, | | | | | | | □ Esquerda | | | | | | | □ Direita | | | | | | □ Ambos | | | | | |  |
| 51. Mão(s) | | | | □ Não | | | | | □ Sim | | | -> Se sim, | | | | | | | □ Esquerda | | | | | | | □ Direita | | | | | | □ Ambos | | | | | |  |
|  | | | | | | | | | | | | | | | | | | | | | | | | | | | | | | | | | | | | | |  |
| **(II)** *Qual dessas dores/queixas é a pior que você sente?________________________________________________*  *Classifique esta dor:* ***Nenhuma dor A pior dor possível***  ***0 1 2 3 4 5 6 7 8 9 10***  **(III)***Você sente alguma delas por mais de três meses?*  □ Não □ Sim -> Se sim, cite qual é esta dor. *__________________________________________________* | | | | | | | | | | | | | | | | | | | | | | | | | | | | | | | | | | | | |  |  |
| **(IV)**  *A partir daqui extremidade superior será usada para representar pescoço, ombro, mão, punho, braço, antebraço e cotovelo.* | | | | | | | | | | | *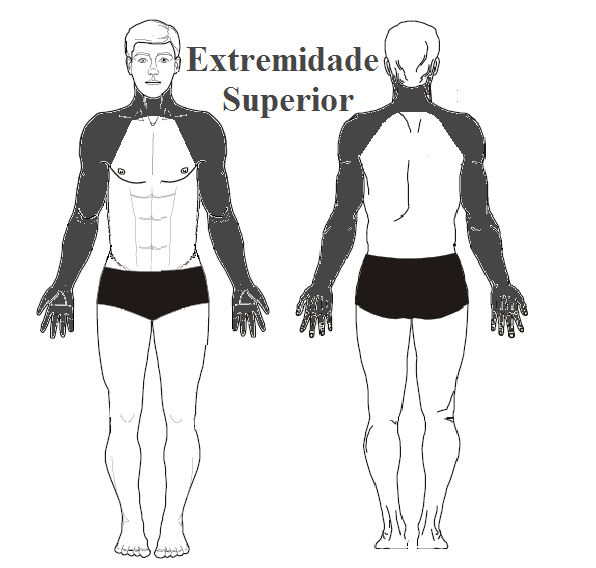* | | | | | | | | | | *Esta imagem mostra as regiões referentes à extremidade superior (em cinza).* | | | | | | | | | | | | | | |  |  |  |
| 52. Durante os últimos três meses eu tive dor/queixa/incapacidade em minha extremidade superior. | | | | | | | | | | | | | | | | | | | | | | | | | | | | | | | | | | | | | |  |
| □ Não | □ Sim | | | | |  | | | | | | |  | | | |  | | | | | | | | | | | | | |  | | | | | | |  |
|  |  | | | | |  | | | | | | |  | | | |  | | | | | | | | | | | | | |  | | | | | | |  |
| 53. O período mais longo de queixa (os últimos três meses) em que eu não pude desenvolver minhas atividades diárias foi: | | | | | | | | | | | | | | | | | | | | | | | | | | | | | | | | | | | | | |  |
| □ Nenhum dia | | | □ ____ Dia(s) | | | | | □ ____ Semana(s) | | | | |  | | | |  | | | | | | | | | | | | | |  | | | | | | |  |
|  | | |  | | | | |  | | | | |  | | | |  | | | | | | | | | | | | | |  | | | | | | |  |
| 54. Durante os últimos três meses eu consultei um médico por causa da dor em minha extremidade superior. | | | | | | | | | | | | | | | | | | | | | | | | | | | | | | | | | | | | | |  |
| □ Não | □ Sim | | | | | -> Se sim, qual foi o diagnóstico médico?________________________________ | | | | | | | | | | | | | | | | | | | | | | | | | | | | | | | |  |
|  |  | | | | |  | | | | | | | | | | | | | | | | | | | | | | | | | | | | | | | |  |
| 55. Que tipo de tratamento você recebeu (os últimos três meses)? | | | | | | | | | | | | | | | | | | | | | | | | | | | | | | | | | | | | | |  |
| □ Nenhum | | □ Fisioterapia | | | | | | □ Medicação | | | | | □ Cirurgia | | | | | | | | | | □ Outro__________________ | | | | | | | | | | | | | | |  |
|  |  | | | | | |  | | | | | |  | | | |  | | | | | |  | | | | | | | | | | | | | | |  |
| 56. Por causa da dor em minha extremidade superior eu já perdi algum emprego. | | | | | | | | | | | | | | | | | | | | | | | | | | | | | | | | | | | | | |  |
| □ Não | □ Sim | | | | |  | | | | | | |  | | | |  | | | | | | | | | | | | | |  | | | | | | |  |
| 57. Por causa da queixa em minha extremidade superior (nos últimos três meses) eu faltei no trabalho. | | | | | | | | | | | | | | | | | | | | | | | | | | | | | | | | | | | | | |  |
| □ Não | □ Sim | | | | |  | | | | | | |  | | | |  | | | | | | | | | | | | | |  | | | | | | |  |
|  |  | | | | |  | | | | | | |  | | | |  | | | | | | | | | | | | | |  | | | | | | |  |
| 58. Durante os últimos três meses, as minhas atividades foram prejudicadas por causa das minhas queixas na extremidade superior. | | | | | | | | | | | | | | | | | | | | | | | | | | | | | | | | | | | | | |  |
| □ Não | □ Sim | | | | | □ Em meu trabalho | | | | | | | | □ Em meu tempo de lazer | | | | | | | | | | | | | | | | |  | | | | | | |  |
|  |  | | | | |  | | | | | | |  | | | |  | | | | | | | | | | | | | |  | | | | | | |  |
| 59. Minhas queixas são devido a um acidente prévio. | | | | | | | | | | | | | | | | | | | | | | | | | | | | | | | | | | | | | |  |
| □ Não | □ Sim | | | | |  | | | | | | |  | | | |  | | | | | | | | | | | | | |  | | | | | | |  |
| **(V)** *As próximas questões estão relacionadas às queixas de dor na extremidade superior nos últimos três meses.* | | | | | | | | | | | | | | | | | | | | | | | | | | | | | | | | | | | | | |  |
|  |  | | | | |  | | | | | | |  | | | |  | | | | | | | | | | | | | |  | | | | | | |  |
| 60. Eu sinto dor em meu membro superior logo que termino o trabalho. | | | | | | | | | | | | | | | | | | | | | | | | | | | | | | | | | | | | | |  |
| □ Não | □ Sim | | | | | -> Esta dor desaparece após um curto período de descanso? | | | | | | | | | | | | | | | | | | | | | □ Não | | | | | | □Sim | | | | |  |
|  |  | | | | |  | | | | | | |  | | | |  | | | | | | | | | |  | | | | | |  | | | | |  |
| 61. Eu sinto fadiga e exaustão em minha extremidade superior. | | | | | | | | | | | | | | | | | | | | | | | | | | | | | | | | | | | | | |  |
| □ Não | □ Sim | | | | | -> Esta queixa desaparece após um curto período de descanso? | | | | | | | | | | | | | | | | | | | | | □ Não | | | | | | □Sim | | | | |  |
|  |  | | | | |  | | | | | | |  | | | |  | | | | | | | | | |  | | | | | |  | | | | |  |
| 62. Eu sinto rigidez em meus dedos. | | | | | | | | | | | | | | | | | | | | | | | | | | | | | | | | | | | | | |  |
| □ Não | □ Sim | | | | | -> Esta rigidez desaparece após um curto período de descanso? | | | | | | | | | | | | | | | | | | | | | □ Não | | | | | | □Sim | | | | |  |
|  |  | | | | |  | | | | | | |  | | | |  | | | | | | | | | |  | | | | | |  | | | | |  |
| 63. Eu sinto dormência em meus dedos. | | | | | | | | | | | | | | | | | | | | | | | | | | | | | | | | | | | | | |  |
| □ Não | □ Sim | | | | | -> Esta dormência desaparece após um curto período de descanso? | | | | | | | | | | | | | | | | | | | | | □ Não | | | | | | □Sim | | | | |  |
|  |  | | | | |  | | | | | | |  | | | |  | | | | | | | | | |  | | | | | |  | | | | |  |
| 64. Eu sinto formigamento em meus dedos. | | | | | | | | | | | | | | | | | | | | | | | | | | | | | | | | | | | | | |  |
| □ Não | □ Sim | | | | | -> Este formigamento continua após o trabalho? | | | | | | | | | | | | | | | | | | | | | □ Não | | | | | | □Sim | | | | |  |
|  |  | | | | |  | | | | | | |  | | | |  | | | | | | | | | |  | | | | | |  | | | | |  |
| 65. Eu sinto fraqueza em minha extremidade superior. | | | | | | | | | | | | | | | | | | | | | | | | | | | | | | | | | | | | | |  |
| □ Não | □ Sim | | | | | -> Esta fraqueza continua após o trabalho? | | | | | | | | | | | | | | | | | | | | | □ Não | | | | | | □Sim | | | | |  |
|  |  | | | | |  | | | | | | |  | | | |  | | | | | | | | | |  | | | | | |  | | | | |  |
| 66. Minhas mãos incham. | | | | | | | | | | | | | | | | | | | | | | | | | | | | | | | | | | | | | |  |
| □ Não | □ Sim | | | | | -> Este inchaço continua após o trabalho? | | | | | | | | | | | | | | | | | | | | | □ Não | | | | | | □Sim | | | | |  |
|  |  | | | | |  | | | | | | |  | | | |  | | | | | | | | | |  | | | | | |  | | | | |  |
| 67. Eu sinto inchaço/rigidez em minha extremidade superior. | | | | | | | | | | | | | | | | | | | | | | | | | | | | | | | | | | | | | |  |
| □ Não | □ Sim | | | | |  | | | | | | |  | | | |  | | | | | | | | | | | | | |  | | | | | | |  |
|  |  | | | | |  | | | | | | |  | | | |  | | | | | | | | | | | | | |  | | | | | | |  |
| 68. Eu sinto uma dor contínua em minha extremidade superior. | | | | | | | | | | | | | | | | | | | | | | | | | | | | | | | | | | | | | |  |
| □ Não | □ Sim | | | | |  | | | | | | |  | | | |  | | | | | | | | | | | | | |  | | | | | | |  |
|  |  | | | | |  | | | | | | |  | | | |  | | | | | | | | | | | | | |  | | | | | | |  |
| 69. Eu percebo mudança de cor, temperatura e transpiração em minha extremidade superior. | | | | | | | | | | | | | | | | | | | | | | | | | | | | | | | | | | | | | |  |
| □ Não | □ Sim | | | | |  | | | | | | |  | | | |  | | | | | | | | | | | | | |  | | | | | | |  |
|  |  | | | | |  | | | | | | |  | | | |  | | | | | | | | | | | | | |  | | | | | | |  |
| 70. Dentre os seguintes itens, quais você utiliza para reduzir sua dor relacionada ao trabalho? | | | | | | | | | | | | | | | | | | | | | | | | | | | | | | | | | | | | | |  |
| □ Nenhum | | | □ Munhequeira | | | | | | | □ Cinta | | | □ Colar Cervical | | | | | | | | | | □ Outro__________________ | | | | | | | | | | | | | | |  |
|  | | |  | | | | | | |  | | |  | | | |  | | | | | | | | | | | | | |  | | | | | | |  |
| 71. Dentre os seguintes itens, quais você utiliza para reduzir sua dor relacionada ao trabalho? | | | | | | | | | | | | | | | | | | | | | | | | | | | | | | | | | | | | | |  |
| □ Nenhum | □ Suporte para mouse | | | | | | | | □ Apoio para pés | | | | | | □ Suporte para textos | | | | | | | | | □ Outro______________ | | | | | | | | | | | | | |  |
